# Reports of the Trustees, Steward, Treasurer and Superintendant of the Insane Hospital, 1844

**Published:** 1845-04

**Authors:** 


2. *Reports of the Trustees, Steward, Treasurer and Superintendent of the Insane Hospital*, 1844. Augusta, (Me.,) 1845.

